# 

**Published:** 1902-12-13

**Authors:** 


**CADBURY'S COCOA** ABSOLUTELY PURE.

**CADBURY'S COCOA** NO DRUGS OR ALKALIES USED.

### THE HOSPITAL

LIBRARY [illegible] 1902

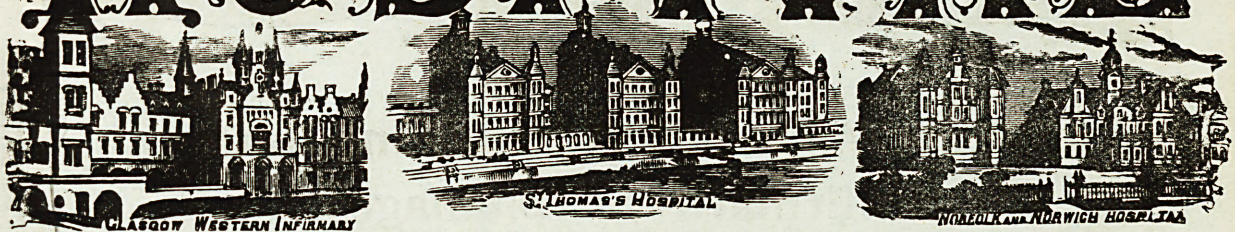

No. 846. Vol. XXXIII. [Registered as a Newspaper.] SATURDAY, DEC. 13TH, 1902. [Subscription Terms, see p. 174] PRICE TWOPENCE.

### WITH SPECIAL CHRISTMAS SUPPLEMENT.

